# Detection of Clenbuterol Hydrochloride Residuals in Pork Liver Using a Customized Surface Plasmon Resonance Bioanalyzer

**DOI:** 10.1371/journal.pone.0122005

**Published:** 2015-03-23

**Authors:** Jiandong Hu, Ruipeng Chen, Shun Wang, Tingting Wang, Yuanyuan Zhao, Jianwei Li, Xinran Hu, Hao Liang, Juanhua Zhu, Xiaohui Sun, Liuzheng Ma, Min Jiang

**Affiliations:** 1 Department of Electrical Engineering, Henan Agricultural University, Zhengzhou, China; 2 State key laboratory of wheat and maize crop science, Zhengzhou, China; 3 Hanan Mechancial and Electrical Vocational College, Zhengzhou, China; 4 School of Human Nutrition and Dietetics, McGill University, Ste Anne de Bellevue, Quebec, Canada; 5 Department of Electronic and Telecommunications, University of Gavle, Gävle, Sweden; 6 College of life sciences, Henan Agricultural University, Zhengzhou, China; University of Akron, UNITED STATES

## Abstract

A surface plasmon resonance (SPR) immunoassay with an immobilization of self-assembled molecular identification membrane for the detection of residual Clenbuterol Hydrochloride (CLB) in pork liver was systematically investigated and experimentally validated for its high performance. SPR immunoassay with a regular competitive inhibition assay cannot be directly verified to detect CLB residuals. In this study, the binding of Au film with mercaptopropionic acid was investigated using the known form of the strong S-Au covalent bonds formed by the chemical radical of the mercaptopropionic acid and the Au film. After that, the immunoglobulin IgG of swine (SwIgG-CLB) was bonded with the mercaptopropionic acid by covalent -CO-NH- amide bonding. The modified comprehensive analysis of how the membrane structure works was introduced together with the customized SPR bioanalyzer. In order to evaluate the performance of this biomembrane structure, the concentrations of CLB-contained solutions of 0 ng•mL^-1^, 10 ng•mL^-1^, 20 ng•mL^-1^, 33.3 ng•mL^-1^, and 40 ng•mL^-1^ were prepared by adding CLB reagents into the solutions of CLB antibody (Clenbuterol Hydrochloride Antibody, CLB-Ab), successively and then the response unit (RU) was measured individually. Using the data collected from the linear CCD array, the fitting curve was established with the R-Square value of 0.9929. Correspondingly, the recovery rate ranged from 88.48% to 103.21% was experimented and the limit of detection of CLB in 1.26 ng•mL^-1^ was obtained efficiently. It was concluded that the detection method associated with biomembrane properties is expected to contribute much to the determination of residual CLB in pork liver quantitatively by using the customized SPR bioanalyzer.

## Introduction

Clenbuterol Hydrochloride (**CLB**) is 1-(4-amino-3,5-dichlorophenyl)-2-(tert-butylamino) ethanol hydrochloride. Clenbuterol is a β_2_ agonist with some structural and pharmacological similarities to epinephrine and salbutamol, but its effects are more potent and longer-lasting as a stimulant and thermogenic drug. It causes an increase in aerobic capacity, central nervous system stimulation, and an increase in blood pressure and oxygen transportation by promoting protein synthesis [1,2]. At the end of the 1970s, The United States of America and other developed countries started to study on practical applications of CLB, which was used as an agent of nutrition redistribution and a growth promoting agent [3]. In the 1980s, it was widespread applied to livestock and poultry production. However the data demonstrated that it would emerge residual CLB in animal tissue and would damage human health by means of the food chain. In recent years, poisoning events that were caused by eating pork contained CLB were repeatedly reported in many countries. Now CLB is illegally used as a growth promoter in animal production. As a result, CLB had become banned drugs in the worldwide pig breeding.

Several approaches for the detection of CLB in liver matrix and urine have been developed [4,5]. For instance, high performance liquid chromatographic (**HPLC**), liquid chromatography-mass spectrometry (**LC-MS**), gas chromatography-mass spectrometry (**GC-MS**) and Enzyme-linked immunosorbent assay (**ELISA**) have been intensively utilized for the quantification and confirmation of CLB [6,7]. Moreover, the immunochromatographic lateral flow test strip and the carbon nanotube are found to be the rapid approaches for the detection of CLB [8–10]. In the last decade, the remarkable developments were exhibited in this field of optical biosensors. Surface plasmon resonance (**SPR**) biosensor for the detection of multi β-agonist residues in liver matrix was preliminarily reported ten years ago. A biological detection approach using an optical SPR biosensor has been developed to detect CLB using the molecularly imprinted membrane [11,12]. The ultrasensitive detection of clenbuterol by quantum dots based electroluminescent immunosensor and an electrochemical immunosensor for the rapid determination of clenbuterol by using magnetic nanocomposites to modify the screen printed carbon electrode have been investigated [13,14]. Furthermore, the detection of residual ractopamine by SPR-based biosensor with an inhibition immunoassay has been studied [15,16]. Comparing with the traditional biochemical analysis method, SPR biosensor is a label-free, non-destructive method for quantitative biological analysis [17,18]. As a result, the SPR biosensor used for the detection of biological samples has been successfully applied in food safety, environment monitoring, drug screening and biomedicine [19,20]

This paper effectively offers a comprehensive analysis of how the membrane structure works. The preparation of the novel biomolecular recognition membrane was performed by the CLB conjugated with the immunoglobulin IgG of swine (**SwIgG-CLB**) instead of the commercially carbo-xymethylated dextran chip. The process of the specific association and dissociation between CLB and SwIgG-CLB was monitored by this SPR biosensor efficiently, which was related to the novel molecular recognition membrane.

## Materials and Methods

### Materials

CLB was obtained from sigma-aldrich (USA). CLB conjugated with the immunoglobulin IgG of swine (SwIgG-CLB) and Clenbuterol Hydrochloride Antibody (CLB-Ab) are provided by Henan Academy of Agricultural Sciences. Mercaptopropionic acid (MPA), 1-Ethyl-3-(3-dimethylaminopropyl) carbodiimide (EDC), N-hydroxysuccinimide (NHS), Sodium dodecyl sulfate (SDS), Ethanolamine (Eth), Phosphate buffer solution (PBS, pH 7.4), 15% (w/v) Sodium carbonate solution, Ethyl acetate, Propyl alcohol, 0.2 mol/L HCL, 0.1mol/L NaOH, Ammonium acetate (pH 5.2) and Glycine were obtained from TCI Development Co., Ltd (Shanghai, China). The three-channel integrated biosensors were purchased from Nomadics, Inc. (Stillwater, USA). The 50nm Au film is deposited on the surface of a BK7 glass slide with a dimension of 18mm×10mm×0.5mm by using a vacuum sputtering coating machine. It has been customized from Beijing JJAM Co. Ltd. (Beijing, China).

All procedures were approved by Henan Agricultural University Animal Care and Use Committee.

CLB-containing samples were collected by Key Laboratory for Animal-derived Food Safety of Henan Province affiliated to College of Animal Husbandry and Veterinary, Henan Agricultural University, China and transferred to us. The samples were collected as part of standard veterinary care, complying with the standard procedures issued by Henan Agricultural University Animal Care and Use Committee.

### Construct of the SPR biosensing system for the detection of CLB residuals

**Fig 1:**
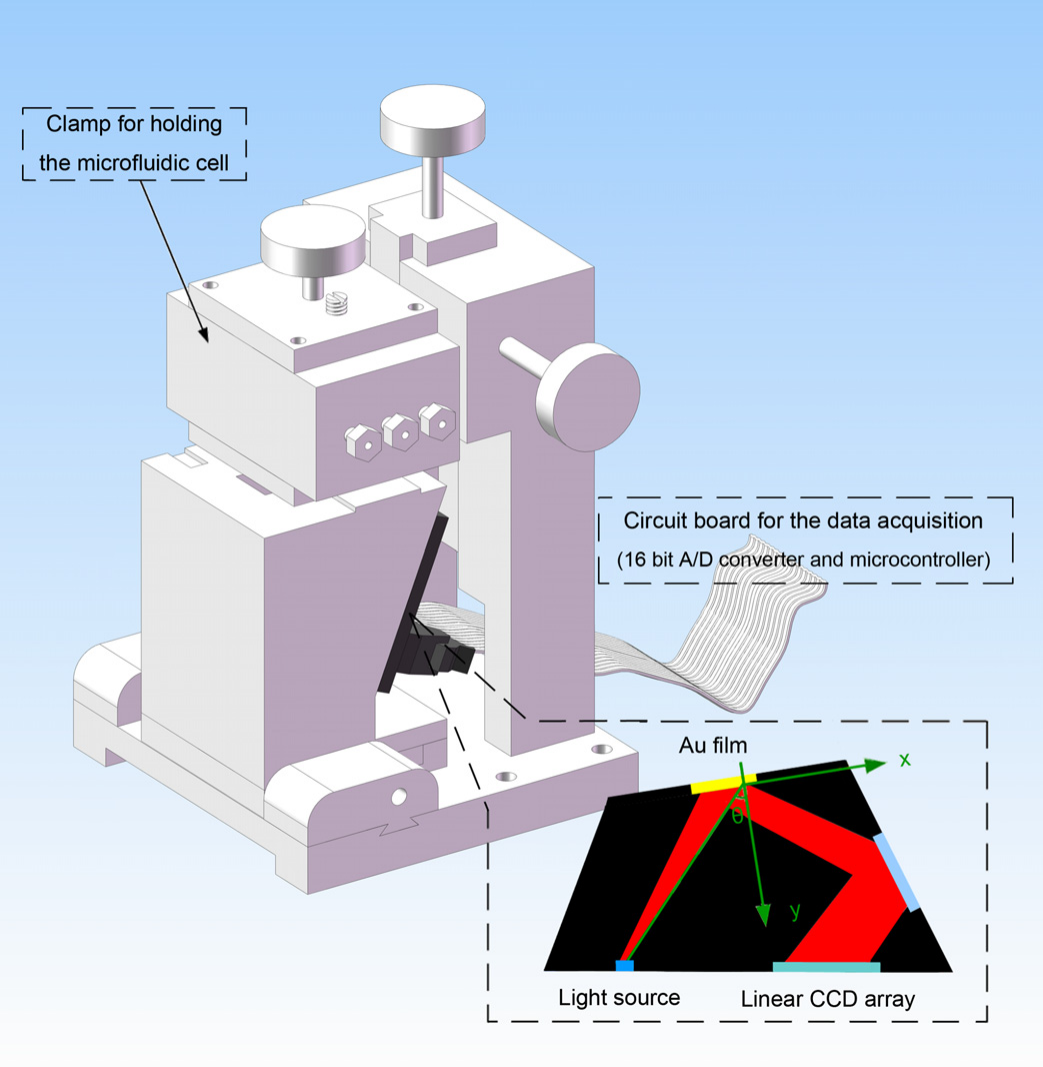
The schematic diagram of the customized SPR biosensing system for the detection of CLB including the clamp for holding the microfluidic cell, the SPR biosensor, the specific support frame and the circuit board for data acquisition.

The novel SPR biosensing system is mainly composed of the integrated biosensor module (Stillwater, USA) and the home made microfluidic cell. Correspondingly, a specified clamp and the electronic control circuit board were developed for loading and unloading the integrated biosensor conveniently and for collecting the response signals from the CCD (**Charge Coupled Device**), respectively. SPR signal changes with refractive index on the Au film surface, in addition, the change of refractive index is proportional to biological molecular mass binding on the metal surface, thus specific signal of biomolecular interaction can be obtained through dynamic changes of biological reaction process of SPR angle. This unique block was easily obtained by packaging with a dark plastic board, which is indicated in Fig. 1. The clamp for holding the microfluidic cell, the SPR biosensor and the specific support frame of this biosensing system are included in this unique block. The change relationship between the resonant dip and the refractive index changed over the biosensor surface, which is caused by the binding of analyte to a receptor immobilized on the biosensor surface, is strongly proportional.

From Fig. 1, it is known that the evanescent wave produced from the total internal reflection on the high refractive index substrate can excite a standing charge density wave on the gold surface [21, 22]. A surface plasmon wave will be generated by standing charge density wave at the interface between the metal film and biological medium, which is a P-polarized electromagnetic wave due to P-polarized light paralleling to incident plane. However, S-polarized light is perpendicular to incident plane.

For the biosensor constructed by a prism with the coupling method of attenuated total reflection, Propagation constants of the incident light wave and the surface plasmon wave along the x axis will be obtained in equation 1 and equation 2.

$$K_{x}^{pr}=\sqrt{\varepsilon_{pr}}\frac{\omega }{c}\sin\theta_{pr}$$ (1)

$$K_{x}^{sp}=\sqrt{\frac{{\tilde{\varepsilon }}_{m}\varepsilon_{s}}{{\tilde{\varepsilon }}_{m}+\varepsilon_{s}}}\frac{\omega }{c}$$ (2)

$$K_{x}^{pr}=\sqrt{\varepsilon_{pr}}\frac{\omega }{c}\sin\theta_{pr}=\sqrt{\frac{{\tilde{\varepsilon }}_{m}\varepsilon_{s}}{{\tilde{\varepsilon }}_{m}+\varepsilon_{s}}}\frac{\omega }{c}=K_{x}^{sp}$$ (3)

where the propagation constants for incident light wave and the surface plasmon wave are indicated with $k_{x}{}^{pr}$ and $K_{x}^{sp}$ respectively. ${\tilde{\varepsilon }}_{m}$ is the complex refractive index of the metal film. *θ*_*pr*_is the angle formed between the incident light and the normal line of the prism. *ε*_*s*_ is the refractive index of the biological sample flowed through the metal film surface. C is speed of light and omega *ω* is the frequency of the surface plasmon wave.

Both propagation constants will be equal, $K_{x}{}^{sp}=K_{x}{}^{pr}=K_{x}$ while the surface plasmon resonance phenomenon is occurring. At the resonance point, the intensity of the incident light is absorbed greatly. The intensity of reflective light is approximately zero. The refractive index of the biological sample bound on the surface of gold film will be calculated by using the equation 3. This is seen by a minimum intensity value in the reflection spectra. The position of the minima is indicative of the biological analytes on the surface of the SPR sensor. The working temperature for this biosensor is controlled to be 25±0.5°C to keep the biosensor working effectively [17]. The home-made three-channel microfluidic cell (2.5μL) was embedded on the surface of the biosensor. The data from the linear CCD were sent out via the Universal Serial Bus (USB) interface to the upper computer.

### Fabrication and functionalization of biomolecular recognition membrane

**Fig 2:**
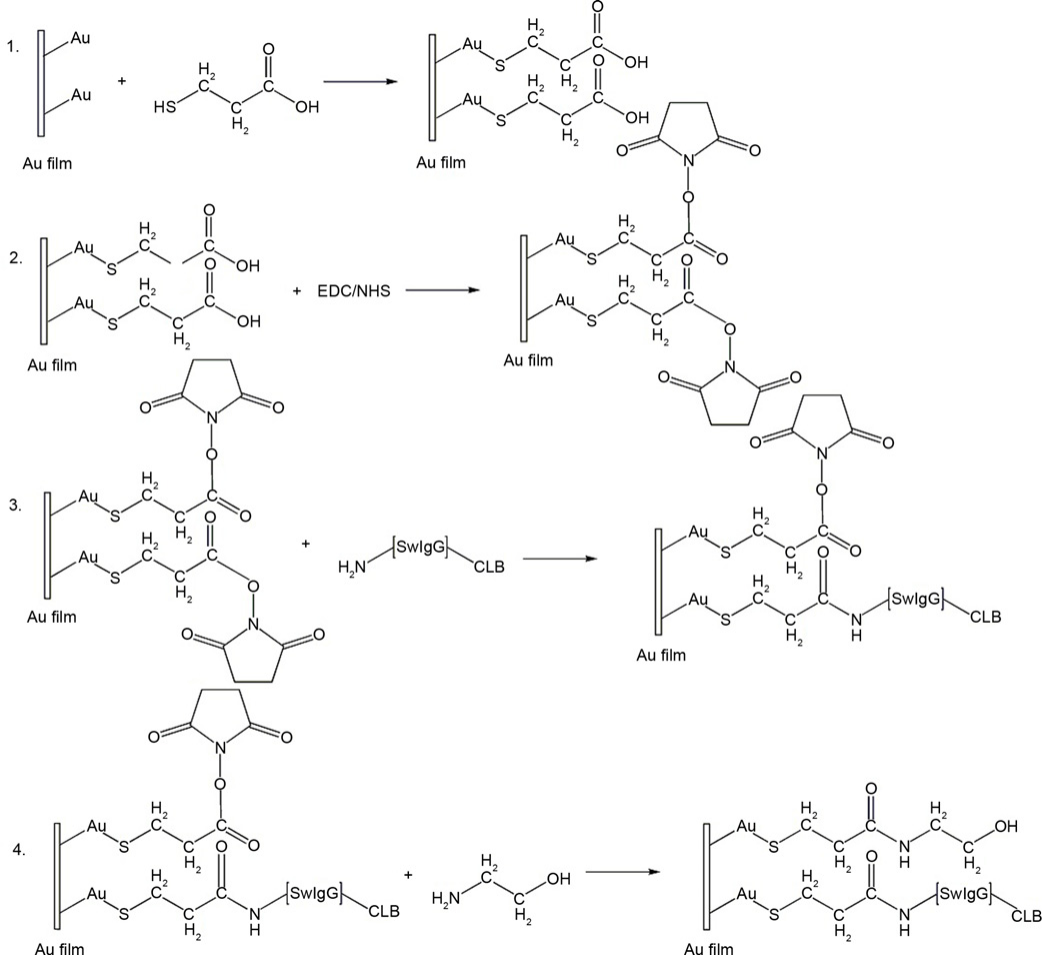
The structure of the biomolecular recognition membrane for the detection of CLB.

Functionalization is the process that the molecular recognition membrane is prepared completely for the detection of CLB using SPR immunoassay. The changes of refractive index are too small if the CLB-Ab is directly immobilized on the Au film surface of the biosensor due to the low molecular weight of CLB. The effect of the direct detection will be unsatisfied because the shift of resonance dip on the linear CCD is small. Therefore, the SPR with competitive inhibition assay cannot be directly used to detect CLB. In this experiment, CLB conjugated with the immunoglobulin IgG of swine (SwIgG-CLB) was used to overcome the above difficulty. The MPA solution is injected to make the-SH group couple with Au film deposited on the surface of the biosensor completely in order to immobilize the synthesis of SwIgG-CLB. Then the immobilization of SwIgG-CLB onto the Au film surface of the biosensor is accomplished by covalent-CO-NH- amide bonding. (1) the biosensor should be cleaned by soaking it into a 4% (v/w) SDS solution to rinse in an ultrasonic bath for 15min and then cleaned with deionized water, dry it with N_2_. (2) the PBS was injected through the microfluidic cell over the surface of the biosensor to obtain the stable RU. (3) the Au film of the biosensor was modified by 1.0 mol·L^-1^ of MPA solution to form a self-assembled monolayer (SAM) for 2h. In order to wash away the unbound MPA, the PBS was injected through the microfluidic cell. (4) the mixture of 0.4 mol·L^-1^ EDC/0.1 mol·L^-1^ NHS (1:1,v/v) was injected through the Au film surface to active the carboxyl groups of the self-assembled monolayer for 1h. Accordingly, PBS was used to wash away residual EDC/NHS. (5) SwIgG-CLB which reacts with carboxyl groups of MPA to form amides was flowed over the SAM surface. The unbound SwIgG-CLB should be washed away by PBS. (6) Eth was injected to confirm the carboxyl sealing off for 20 min. There is a competition relationship between the CLB molecules in the samples and the SwIgG-CLB on the surface of the biosensor, as both of them can be combined with the antibody in the samples. The concentration of CLB is inversely proportional to the response signal. When the concentration of CLB in the sample is small, more antibodies can be combined with the biomolecular recognition membrane on the surface of the biosensor [22]. The structure of the biomolecular recognition membrane for the detection of CLB is shown in Fig. 2.

### Sample preparation

The homogenized liver samples were extracted from the pork then 5g liver sample was taken into a 50 ml microcentrifuge tube labeled with A. 20 ml ethyl acetate and 2 ml of 15% (w/v) sodium carbonate solution were added into the microcentrifuge tube A. It is vibrated by using an ultrasonic oven for 15 min. It is then centrifuged at a speed of 6000 rpm (**Revolutions per minute**) for 5 min at the temperature of 4°C. The supernatant is transferred into the 15 mL microcentrifuge tube labeled with B. After that, 10ml ethyl acetate is added into the microcentrifuge tube A and it is vibrated by using the ultrasonic oven for 15min. The supernatant is transferred into the microcontrifuge tube B and well mixed with the original supernatant. 10 ml propyl alcohol is added into the microcontrifuge tube B and it is concentrated by using the rotary evaporator with the water bath at 50°C to complete dryness. 20 ml ethyl acetate is added into the microcontrifuge tube B to dissolve the supernatant. It is mixed with a 2 mL 0.2 mol/L HCL solution and centrifuged at a speed of 5000 rmp for 5 min in room temperature. The supernatant is transferred out into a graduated microcentrifuge tube labeled C and the solution left in the microcentrifuge tube B is extracted again. The new supernatant is well mixed with the supernatant in the graduated microcentrifuge tube C. The 0.1mol/L NaOH is added into it to obtain the pH value 5.2 of the supernatant solution and then 20 mmol/L ammonium acetate (pH 5.2) is used to reach a constant marked volume of 10mL. After that, the analytical solution has been prepared.

### Detection of CLB residuals in pork liver

In this experiment, the different concentrations of standard CLB ranged from 0 ng·mL^-1^ to 40 ng·mL^-1^ was injected through the microfluidic cell over the molecular recognition membrane for 10 min, followed by a thorough rinsing with glycine to dissociate the nonspecific bindings. The averaged results from repeated experiments were calculated to reduce the noises embedded in the photoelectric signals from the CCD array. The detection of CLB residuals was performed by monitoring the changes of response units with time [19].

For the evaluation of reproducibility of this special molecular recognition membrane, the known concentration 33.3 ng·mL^-1^ of CLB contained in the analytical sample was flowed over the Au surface with repeated 5 times. A desired amount of exogenous pure CLB reagent was added into the pork liver solutions for validating this approach. A series of pork liver solution were prepared to be measured using this SPR immunoassay. The recovery rate was experimented and calculated.

## Experimental Results and Analysis

### Characteristics of biomolecular recognition membrane for SPR immunoassay

**Fig 3:**
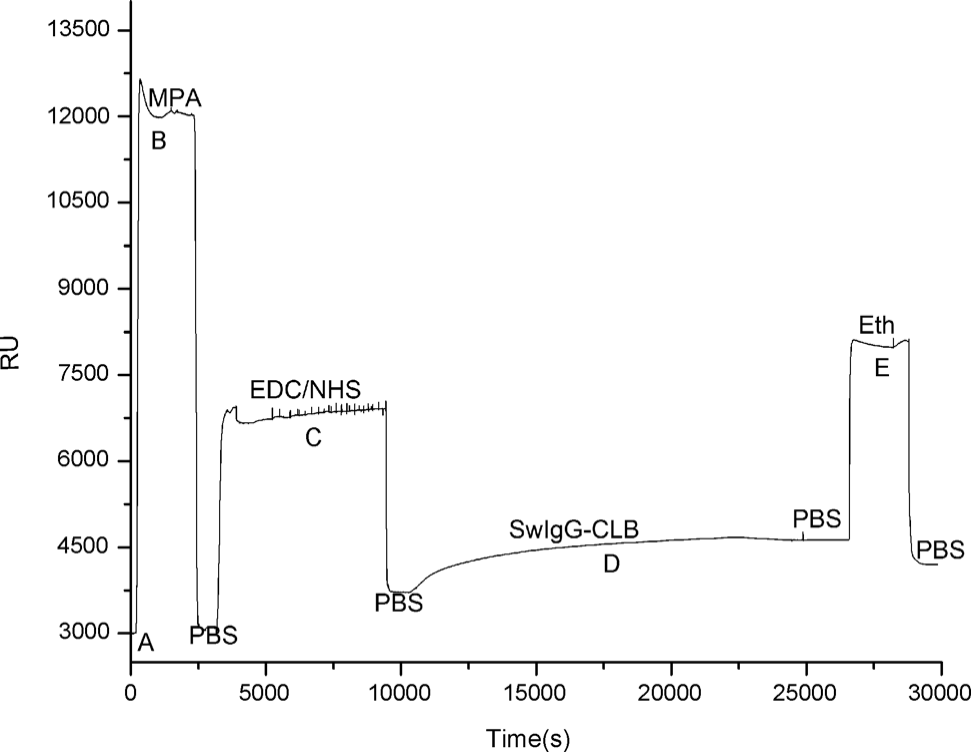
The Response Unit obtained from the functionalization of biomolecular recognition membrane. A: Obtain the baseline by injecting the PBS through the microfluidic cell; B: Form the self-assembled monolayer (SAM) by injecting MPA; C: Activate the carboxyl groups of the self-assembled monolayer by the mixture of EDC/NHS; D: Wash away the unbound SwIgG-CLB by PBS; E: Seal off the carboxyl using Eth.

The RU of the biomolecular membrane modification during the preparation is shown in Fig. 3. When PBS was injected by peristaltic pump through the microfluidic cell over the Au film surface of the integrated biosensor at the speed of 30μL·min^-1^, the baseline was set to 3000 RU. The delta Response Unit signal (**ΔRU**, the Response Unit of baseline was subtracted by current Response Unit) obtained from the binding of Au film with MPA was 321 RU. The SwIgG-CLB containing-NH_2_ groups was immobilized by covalent-CO-NH- amide bonding after the carboxyl groups on MPA were activated by the mixed solution of EDC/NHS with the **Δ**RU of 1560 RU (See Fig. 3). From Fig. 3, the response units (RUs) indicated with the baseline is 3688 before the SwIgG-CLB is fixed on the Au film surface of the biosensor, while the values of the RU reach to 4569 at the equilibrium plateau after the antigen of SwIgG-CLB is fixed on the Au surface of the biosensor, then the values of the RUs are obtained steadily to be 4487 after being washed by PBS. The response units only decline a little in RUs, which means the effect of the membrane functionalization of the biosensor is satisfying (See the phase D in Fig. 3). Sequentially, the Eth is flowed over the Au surface of the biosensor to obtain the new equilibrium plateau. The values of the RUs are 4371 after being washing by PBS. The values of RUs in this process are subjected to decrease slightly due to the use of a PBS in an improper pH value. That means the grafting density decreases. However, in this experiment, the actual binding responses are the values obtained by subtracting the background response signals occurred in the reference channel from the response signals produced in the sample channel. Therefore, the sensitivity of the detection would avoid decrease suffering. The effective binding responses are 683 in RU obtained. One RU corresponds approximately to 1 picogram (pg)/mm^2^ of material bound of surface area reported by Chen Situ et al.[23]. The grafting density obtained from the membrane functionalization performed in this experiment is approximately calculated to be 683 pg/mm^2^.

### Quantitative analysis of CLB residuals

The concentration 50 μg·mL^-1^ of CLB-Ab was chosen to be the standard antibody sample. The analytical solutions at different CLB concentrations of 0 ng·mL^-1^, 10 ng·mL^-1^, 20 ng·mL^-1^, 33.3 ng·mL^-1^, and 40 ng·mL^-1^ were obtained respectively. CLB and CLB-Ab were mixed in advance for one night to make sure them reacting sufficiently. First, PBS was injected through the Au film surface to get a stable RU. And then the solution prepared using competitive inhibition assay was flowed through the Au film surface of the biosensor by peristaltic pump, successively to obtain the changes of RU resulted from the changes of the refractive index which was changed by the different concentrations of analytical solutions on the surface of the Au film. Specific binding was emerged between the unreacted CLB-Ab in the sample solution and the SwIgG-CLB immobilized on the Au film surface of the biosensor to generate the sensorgram of antibody and antigen. The sensorgram was displayed on the upper computer.

**Fig 4:**
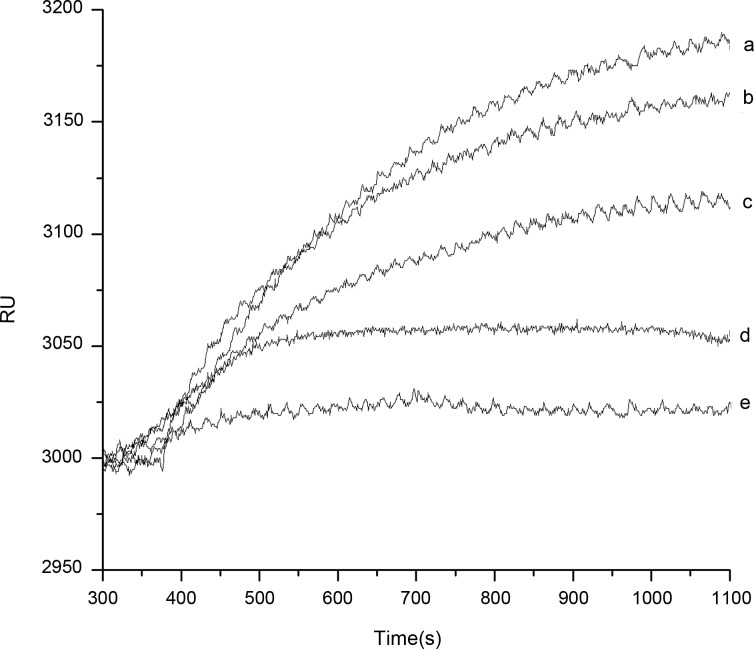
Sensorgram for CLB-Ab binding to the immobilized SwIgG-CLB surface. The known concentrations of CLB in sample solution are shown at the right of each sensorgram, indicating with a, b, c, d, e corresponding to the concentrations of 0 ng·mL^-1^, 10 ng·mL^-1^, 20 ng·mL^-1^, 33.3 ng·mL^-1^ and 40 ng·mL^-1^, respectively.

Fig. 4 shows the association process between the analytical sample at different CLB concentrations of 0 ng·mL^-1^, 10 ng·mL^-1^, 20 ng·mL^-1^, 33.3 ng·mL^-1^ and 40 ng·mL^-1^ and the SwIgG-CLB immobilized on the surface of the Au film. The interactional sensorgram was generated after the analytical samples were flowed through the Au film surface. The ΔRU obtained by the Response Units subtracted to the baseline value were 190, 140, 112, 58, and 30. The ΔRU decreases with the amount of CLB increasing, which agrees with the rules of the modified competitive inhibition immunoassay and had preferable discrimination between each concentration.

**Fig 5:**
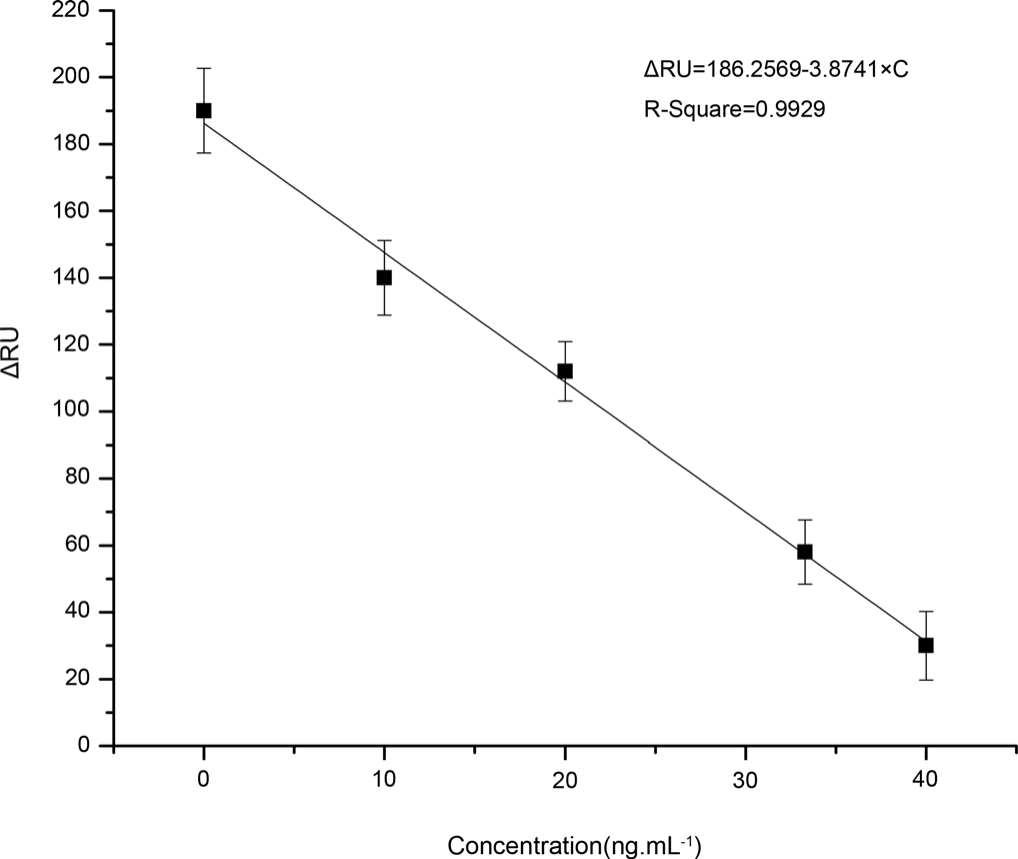
The fitting curve of the delta Response Units with different standard CLB concentrations.

Using the response results to make curve-fitting, consequently, CLB standard curve was built by utilizing the biosensing membrane. The three repetitive fitting results of each sample were shown in Fig. 5, and the fitting equation Δ*RU* = 1862569–3.8741× *C* was indicated, where ΔRU is the difference between the RU of baseline and the current RU, and C denotes the concentration of CLB in the solution.

The squared correlation coefficient is 0.9929 indicated in the Fig. 5. In order to obtain the theoretic detection limit of CLB, extraordinary, the low concentration sample was used for validation. The limit of detection (**LOD**) was calculated to 1.26 ng·mL^-1^ according to the following expression: LOD = 3σ/b, described in the literature 18, where *σ* is the standard deviation of the analytical sample without CLB and *b* is the slope of the fitting curve.

### Experiment of reproducibility

**Fig 6:**
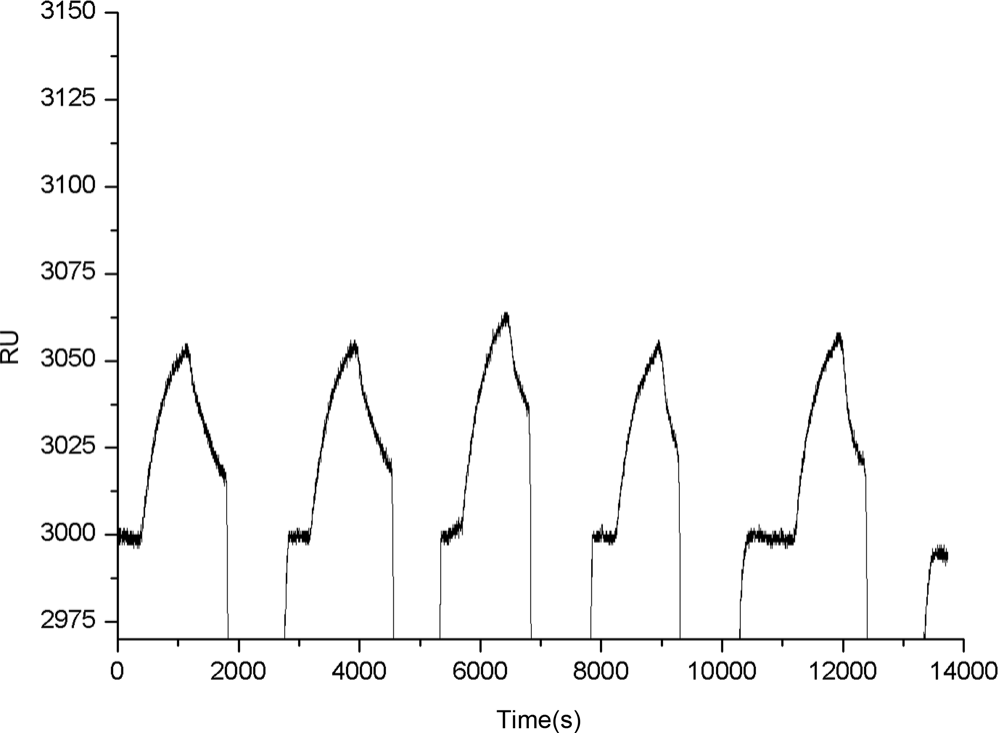
Sensorgram of the known concentration 33.3 ng·mL^-1^ of the CLB sample solution with five repeated measurements.

Reproducibility is essential to reliable validation in the experiment based on SPR biosensor. In this experiment, the known concentration 33.3 ng·mL^-1^ of CLB contained in the analytical sample was flowed over the Au surface repeated 5 times to measure the reproducibility of this approach. The ΔRU of five times repeated measurements were 53, 56, 63, 59 and 61, respectively. The relative standard deviation (RSD) 6.36% was obtained (Fig. 6). In this experiment, we selected the equilibrium plateau as the endpoint for calculating the ΔRU. The equilibrium plateau is considered to be reached when the response signal to time ratio dRU/dt is less than 0.2 (changes in the value of RU within 5 min are less than 1). The results obtained from the reproducibility experiment proved that the approach is reasonable in a certain situation for detecting CLB contained in sample.

### Recovery experiment

In order to investigate the detecting efficiency of this novel biomembrane structure, a recovery study was realized. For the CLB detection based on SPR, the recovery of the analytical sample is the difference between the measured concentration of CLB from the fitting equation and the actual concentration of CLB added in the analytical sample. The recovery rate is then calculated from the following expression: Recovery rate = C_1_/C_0_×100%, where C_0_ is the actual concentration of CLB and C_1_ is the measured concentration of CLB calculated from the fitting equation.

The process was detailed as follows: take 500μL solution with 10 folds dilution which was prepared from the pork liver to the four clean centrifuge tubes, respectively and mark it as the comparison sample without CLB. The ΔRU were measured as 180, 165, 151, and 158 respectively by using this novel approach. As the procedures described above, the concentration 33.33 ng·mL^-1^ of CLB obtained after adding the CLB into the prepared sample solutions were marked as the analytical samples. The four measurement results of the ΔRU were obtained as 108, 102, 98, and 104 by using the competitive inhibition assay.

The recoveries were obtained by the standard-addition method ranged from 88.48% to 103.21%, indicating the influence of background after four repeated measurements (See Table 1).

**Table 1:** Measurement results of the recovery rate

Number of Measurements	Before added CLBΔRU_1_ ^1^	After added CLBΔRU_2_ ^2^	ΔRU = (ΔRU_1_-ΔRU_2_)	The actual concentration, ng/mL	The calculatedconcentration, ng/mL	Recovery rate,%
1	180	108	72	33.33	29.49	88.48
2	165	102	63	33.33	31.82	95.47
3	151	98	53	33.33	34.40	103.21
4	158	104	54	33.33	34.14	102.43
Average	163.5	103	60.5	33.33	32.46	97.40

^1^The ΔRU_1_ is the ΔRU of the prepared sample solution before added the actual concentration of CLB.

^2^The ΔRU_2_ is the ΔRU of the prepared sample solution after added the actual concentration of CLB.

## Conclusions

The novel synthesis of SwIgG-CLB was fixed on the Au film surface of the biosensor to prepare the molecular recognition membrane for the detection of CLB. The CLB-contained samples at the concentration of 0 ng·mL^-1^, 10 ng·mL^-1^, 20 ng·mL^-1^, 33.3 ng·mL^-1^, and 40 ng·mL^-1^ were respectively obtained by adding the CLB reagent into the known concentration of CLB-Ab solution for competition reaction. The performances of the novel biosensing membrane for the detection of CLB in pork liver have been verified by experiments successfully. The linear relationship between the response signals and the residual amount of CLB in sample solutions was obtained with the squared correlation coefficient of 0.9929 and the limit of detection (LOD) of 1.26 ng·mL^-1^, respectively. The clamp for holding the biosensor is a novel design, which can be easily adjusted in various degrees of freedom to align the light beam, the microfluidic channels and the biomolecular recognition membrane.

In conclusion, this is the first detailed investigation of biomembrane structure for the detection of CLB at the lower limit of detection. Furthermore, optical SPR approach for determination of CLB was not only for rapid detection of the residual CLB in pork liver but also on the analysis of the biomolecular interaction between CLB-Ab and CLB. Compared to the routine detection methods, this novel approach with the LOD of 1.26 ng·mL^-1^ is of label-free, small amount of samples (<250μL) and anti-electromagnetic interference characteristics, which provided a novel method of quantitative analysis for the determination of CLB in sample solution.
